# Acute, Nontraumatic Spontaneous Spinal Subdural Hematoma: A Case Report and Systematic Review of the Literature

**DOI:** 10.1155/2017/2431041

**Published:** 2017-12-26

**Authors:** Leigh A. Rettenmaier, Marshall T. Holland, Taylor J. Abel

**Affiliations:** ^1^University of Iowa Carver College of Medicine, 375 Newton Rd, Iowa City, IA 52242, USA; ^2^Department of Neurosurgery, University of Iowa, 200 Hawkins Drive, Iowa City, IA 52245, USA

## Abstract

Spontaneous spinal subdural hematoma (sSDH) is a rare condition outright. Moreover, cases that occur spontaneously in the absence of an identifiable etiology are considerably less common and remain poorly understood. Here, we present the case of a 43-year-old man with spontaneous sSDH presenting with acute onset low back pain and paraplegia. Urgent magnetic resonance imaging identified a dorsal SDH from T8 to T11 with compression of the spinal cord. Emergent T8–T10 laminectomies with intradural exploration and hematoma evacuation were performed. However, despite prompt identification and appropriate action, the patient's recovery was modest and significant disability remained at discharge. This unique and unusual case demonstrates that spontaneous sSDH requires prompt surgical treatment to minimize associated morbidity and supports the association between the presence of severe neurological deficits upon initial presentation with less favorable outcomes. We performed a comprehensive systematic review of spontaneous sSDH of unknown etiology, which demonstrates that emergent surgical intervention is indicated for patients presenting with severe neurological deficits and the presence of these deficits is predictive of poor neurological outcome. Furthermore, conservative management should be considered in patients presenting with mild neurological deficits as spontaneous resolution followed by favorable neurological outcomes is often observed in these patients.

## 1. Introduction

Although spontaneous spinal subdural hematoma (sSDH) is a rare condition, it is associated with significant morbidity and mortality [1]. Exceedingly less common are spontaneous sSDHs occurring in the absence of an identifiable etiology. A nearly equivalent incidence between males and females has been described, but given the rarity of spontaneous sSDH the exact incidence remains unknown [2]. While spontaneous sSDHs are most frequently described in association with coagulopathies, iatrogenic causes, or arteriovenous malformations [1], the pathogenesis of spontaneous sSDH largely remains unclear. Rupture of the vasculature within the subarachnoid or subdural space has been proposed as a potential pathogenic mechanism in certain cases. While some suggest that the bleeding originates from subarachnoid vessels with concomitant rupture into the subdural space following an increase in intra-abdominal or intrathoracic pressure, others have proposed an alternative theory that the bleeding begins in the subdural space itself [3, 4]. Clinical presentation is typified by symptoms representative of spinal cord injury: motor, sensory, and autonomic dysfunction resulting from spinal cord compression [1]. Options for treatment include surgical decompression, percutaneous drainage, or management with conservative therapies alone. In this report, we present the case of a spontaneous sSDH presenting as acute onset lower back pain with paraplegia with no identifiable cause. Given the rarity of this condition, we review the available literature describing spontaneous idiopathic sSDH to elucidate the epidemiology, presentation, pathogenesis, diagnosis, treatment, and outcome of this rare condition.

## 2. Case Report

### 2.1. Presentation

A 43-year-old man presented to the emergency department with acute onset paraplegia and lower back pain that began in the absence of trauma. The patient reported feeling occasional paresthesia in his legs the preceding 2 months; however, the patient had not sought medical evaluation. The night prior to presentation, the patient reported moving quickly to avoid a bar fight that he was not involved in. Following this, he was able to proceed home without any noted difficulty. The following morning, the patient was able to walk, sit, and stand from the sitting position. However, after resting for a period of time, the patient experienced acute onset of unprovoked back pain and noted an inability to move his legs. This prompted emergent medical evaluation.

On examination, the patient was found to have grade 5/5 strength in his bilateral upper extremities and grade 0/5 strength throughout his bilateral lower extremities. He noted normal sensation in his upper extremities and slight decreased sensation in his lower extremities symmetrically. The patient had a postvoid residual of 1,500 cc. He had no history of recent surgical procedures and was not currently taking any prescription or over-the-counter medications.

Initial laboratory data revealed an elevated erythrocyte sedimentation rate of 68 (0–15), elevated C-reactive protein of 3 mg/dL (≤0.5), elevated WBC count of 17,300/*μ*L (3.7–10.5), platelet count of 285,000/*μ*L, partial thromboplastin time of 23 seconds (22–31), prothrombin time of 11 seconds (9–12), and an international normalized ratio of 1.1 (<4.0). Urine drug test was positive for amphetamines, benzodiazepine, and oxycodone.

**Figure 1:**
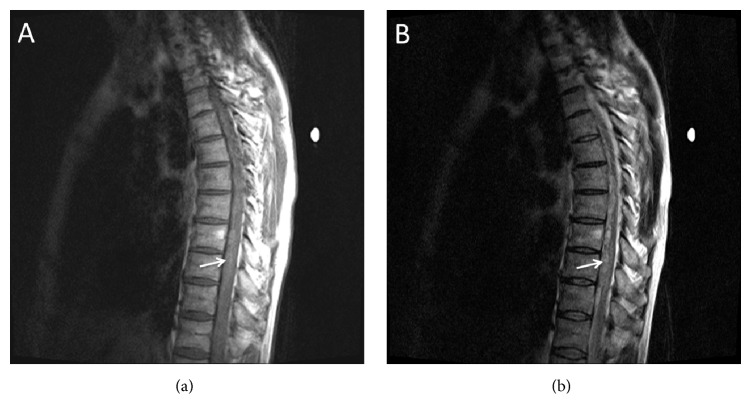
Preoperative MRI sagittal views T1 (a) and T2 (b) of the thoracic spine. White arrow indicates subdural hematoma.

Magnetic resonance imaging (MRI) with and without contrast of the spine was performed. T1- and T2-weighted images revealed an intradural, extramedullary heterogeneous subdural T2 signal and isointense T1 signal located ventral to the spinal cord spanning T8 to T11 causing displacement and compression of the thecal sac consistent with hyperacute or acute subdural hematoma. High T2 signal within the spinal cord at levels T10–T12 demonstrated the presence of spinal cord edema. (See Figure 1). Magnetic resonance angiography (MRA) of the thoracic spine revealed no evidence of arteriovenous malformation or arteriovenous fistula.

### 2.2. Operation

The patient was taken to the operating room emergently for T8–T10 laminectomies, with intradural exploration, and hematoma evacuation. Intraoperatively, a hematoma was visualized upon opening of the thecal sac and the hematoma was evacuated with gentle suction. Following evacuation, the spinal cord was visibly contused and swollen. Otherwise, inspection of the intradural space did not reveal any apparent abnormalities. Specifically, no evidence of abnormal vasculature or masses was observed. Hematoma fragments were collected and sent for histopathologic evaluation.

### 2.3. Postoperative Course and Histopathology

Postoperatively, the patient's initial strength was stable exhibiting grade 0/5 strength in bilateral lower extremities and grade 5/5 strength in bilateral upper extremities. Sensation was unchanged compared to preoperative evaluation. One-week following surgery, the patient's strength showed signs of improvement with grade 3/5 strength in right toe flexion. The patient's recovery was complicated by severe sepsis secondary to* Clostridium difficile* colitis. The patient was discharged on hospital day 25 to an acute rehabilitation facility. At discharge, the patient's examination remained unchanged with grade 3/5 strength in right toe flexion and otherwise 0/5 in all other lower extremity muscle groups and slightly diminished sensation in the bilateral lower extremities. Pathological samples taken at the time of surgery demonstrated acute hematoma with fragments of leptomeninges and meningothelial cells. There was no evidence of a vascular or neoplastic lesion.

Eight weeks following surgery, the patient continued to reside at an inpatient rehabilitation facility. His rehabilitation was complicated by development of a sacral wound requiring incision and drainage and placement of a wound vac. His lower extremity strength improved to consistent grade 2/5 throughout with reported rare ability to move his leg against gravity. His sensation remained stable with decreased (but present) sensation in the bilateral lower extremities. He had no bowel or bladder control using suppositories and self-catheterization techniques.

### 2.4. Review of the Literature

**Figure 2:**
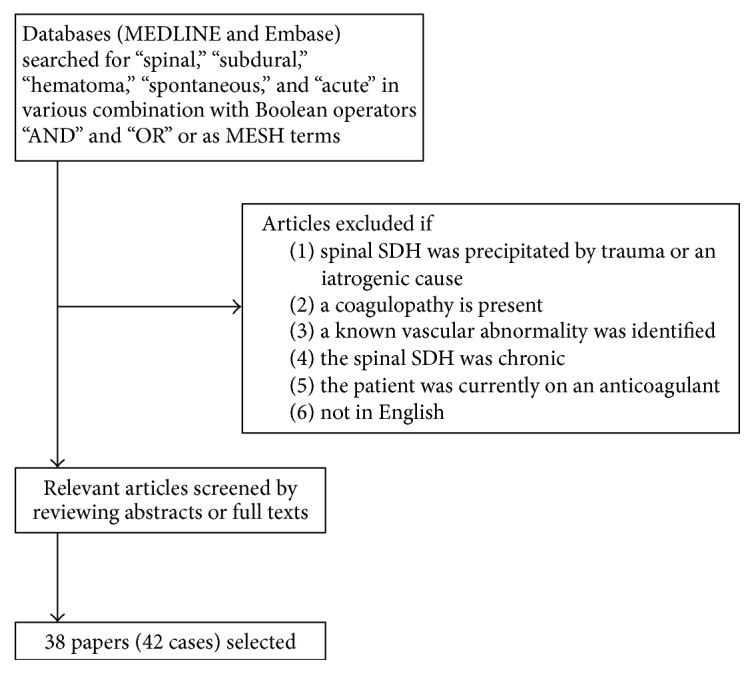
Flow chart detailing search strategy for review of literature.

A review of the English literature was conducted by searching Medline and Embase through November 2016. The terms “spinal subdural hematoma”, “spontaneous spinal subdural hematoma” and “acute spinal subdural hematoma” were used. In a search of Medline, the MESH term “spinal subdural hematoma” returned 108 articles, “spontaneous” and “spinal subdural hematoma” yielded 28 articles, and “acute” and “spinal subdural hematoma” produced 25 articles. In a search of Embase, the terms “spinal hematoma”, “spontaneous,” and “subdural” generated 54 articles. The searches provided 215 papers, which were subsequently reviewed. Papers were excluded if the onset of the sSDH was precipitated by trauma or an iatrogenic cause, if a coagulopathy was present, if a known vascular abnormality was identified, if the sSDH was chronic, or if the patient was currently on an anticoagulant medication. After applying these restrictions, 38 papers were selected and 42 cases were included in the review of the literature (Table 1). Cases were indexed by patient age, gender, presenting symptoms, spinal level, additional medical conditions, treatment/surgical intervention, and patient outcome. Methods for the selection of articles are summarized in Figure 2.

## 3. Results

Forty-three patients with acute spontaneous sSDHs were identified in the review of the literature including the present case. Of the 43 patients, 18 were female, 24 were male, and 1 was unspecified. Patient age ranged from 27 years to 81 years with an average of 53.3 (±14) years. The predominant location of sSDHs was the thoracic spine. Of the 43 patients, 84% (36/43) demonstrated sSDHs spanning the thoracic spine, 23% (10/43) had cervical spine involvement, and 26% (11/43) demonstrated lumbosacral involvement. The location of the sSDH was limited to the cervical spine in only 2 of 43 patients, but 8 additional patients with cervical involvement had extension to the thoracic and/or lumbar regions. The extent of the sSDH ranged from a single level to up to 23 vertebral levels [22]. In 40% (17/43) of cases, the sSDH was limited to 4 or less levels, while 49% (21/43) involved 5 or more levels, and 11% (5/43) were unspecified.

In the review of the literature, back pain or interscapular pain was the most common presenting symptom with 63% (27/43) of patients reporting this as their initial symptom. Neck pain or stiffness was reported in 15% of patients (6/41), while headache was reported in 24% (10/41). Although cases with major bleeding risk factors, such as coagulopathy, were excluded from this study, several patients had additional underlying medical conditions (see Table 1). Of note, hypertension was most commonly encountered, being present in 16% (7/43) of patients identified in this review. Concurrent subarachnoid hemorrhage was described in 4 patients, while concurrent intracranial SDH was reported in 3 patients.

In most cases prior to 1991, myelography was the predominant diagnostic modality for sSDHs, whereas MRI was used in every subsequent case. Spinal angiography was performed in 21 of 43 cases in attempt to identify the source of bleeding. Given our inclusion criteria, no underlying vascular abnormalities were identified in any of the cases. Of the 43 patients, 20 patients (47%) underwent surgical decompression, 22 patients (51%) were managed with conservative therapies only, and 1 patient underwent lumbar puncture with percutaneous drainage of the sSDH. Of those patients managed with conservative therapies only, 86% (19/22) were reported to have either complete or good recovery, while the remaining 14% (3/22) experienced partial recovery. There were no reported cases of poor recovery with conservative therapy. Surgical intervention was employed in the treatment of 20 patients: 45% (9/20) experienced complete or good recovery, 25% (5/20) experienced partial recovery, 20% (4/20) experienced poor or no recovery, and there were 2 patient deaths (1 death was attributed to an unrelated factor). Of the 43 patients, 12 either presented with complete paraplegia or progressed to complete paraplegia shortly after presentation. Of these 12 patients, 8 underwent surgical intervention while the remaining 4 were managed conservatively. Outcomes for patients who underwent surgical decompression included partial recovery (3/8), poor or no recovery (3/8), or death (2/8), although 1 death was attributed to an unrelated factor. Patients presenting with complete paraplegia who were managed conservatively experienced complete or good recovery (50%; 2/4) or partial recovery (50%; 2/4).

## 4. Discussion

### 4.1. Epidemiology

Few publications exist addressing the exact prevalence of spontaneous sSDH; however, it appears to be quite rare. Domenicucci et al. presented a series of 106 cases of nontraumatic acute sSDH; this series reported near equal distribution of males and females with rates of 49% and 51%, respectively [2]. The average age in this series was 47.5 years (rang: 0.5–87 years). Similarly, Pereira et al. described a slight female predominance (1.25 female/1.0 male) in a series of 151 patients with nontraumatic spontaneous acute sSDH [1].

Spontaneous sSDH is most often associated with disorders related to impaired hemostatic mechanisms or following minor injury from iatrogenic causes. In a review of 151 patients with nontraumatic spontaneous acute sSDH, 46% of patients were either treated with anticoagulation therapy or harbored a coagulopathy attributable to a hematologic disorder [1]. In a separate review of 106 cases of nontraumatic acute sSDH, a large proportion of the cases were associated with either bleeding disorders or purely iatrogenic causes, representing 54% and 14% of the cases, respectively [2]. Bleeding disorders were mainly noted as those that impair the hemostatic mechanism including leukemia, hemophilia, thrombocytopenia, cryoglobulinemia, hemorrhagic diathesis, and polycythemia. Although less common, cases of spontaneous sSDH have been reported in the following conditions: ankylosing spondylitis [17, 42], systemic lupus erythematosus [43], fibromuscular dysplasia [24], cystic fibrosis [44], polycystic kidney disease [25], chronic renal failure [34], rhabdomyolysis [31], rheumatoid arthritis [32], pregnancy [45], and eclampsia [46]. Although an underlying coagulopathy, anticoagulant therapy, or an iatrogenic cause can be implicated in most cases of spontaneous sSDH, a significant proportion of patients have no readily identifiable cause; thus further investigation of these cases is warranted.

### 4.2. Presentation

Spontaneous sSDH often presents as acute severe back pain with radicular signs. It is frequently accompanied by sensory, motor, and autonomic dysfunction including erectile dysfunction and urinary retention [1, 14, 20, 39]. Domenicucci et al. reported the most common presenting symptoms to be motor deficits (57% of patients), spinal pain (45% of patients), radicular pain (22% of patients), and paresthesia [2]. Patients may also complain of headache and sphincter dysfunction. The severity of these deficits varies greatly from the presence of only pain without motor or sensory deficits to those of complete quadriplegia [2, 3]. Less common presentations include symptoms of central cord syndrome [12], hemiparesis [28], and initially only headache with neck stiffness [30, 37]. The present case represents a more severe instance with complete paraplegia on initial presentation. What typifies this pathology from an otherwise less worrisome diagnosis is an acute neurological change in the setting of no readily identifiable cause.

### 4.3. Pathogenesis

The pathogenesis of sSDHs is unclear as the bridging veins often implicated in the development of intracranial SDHs are not abundant within the spinal canal [47]. Some have suggested the bleeding in sSDHs results from rupture of vessels within the subarachnoid space following a rapid increase in intrathoracic or intra-abdominal pressure [4]. Any bleeding that originates from the vascular subarachnoid space would be subject to dilution by cerebrospinal fluid, thus preventing hematoma formation within the subarachnoid space. If bleeding within the subarachnoid space becomes sufficiently profuse, it may rupture into the subdural space [48]. Consistent with these propositions, cases in which spinal subarachnoid hemorrhage and SDHs coexist have been reported [3, 12, 23]. Alternatively, rupture of small extra-arachnoid vessels lying along the dural surface may be the source of bleeding in sSDHs [3]. Ultimately, it is difficult to determine whether the source of bleeding originates from within the subarachnoid or subdural space.

Although our patient denied intravenous drug use, a history of drug abuse cannot be excluded, especially considering the positive urine drug test for amphetamines. Two cases of spontaneous sSDH in association with amphetamines have previously been reported [31, 40]. Amphetamine use has been associated with both intracranial hemorrhage and cerebral vasculitis [49]. Although a direct causal link cannot be made, amphetamine use may have contributed to the development of sSDH in the present case considering the absence of other definitive contributing factors and the suggested mechanisms relating amphetamine use to vascular pathologies.

### 4.4. Diagnosis

MRI is considered the gold standard in the evaluation of sSDHs as it is capable of visualizing spinal hematomas as well as other spinal cord pathologies. The appearance of the sSDH on MR imaging is dependent on its duration and oxygenation and has been previously described [22]. Prior literature has shown that contrast-enhanced time-resolved MR angiography was 88% sensitive, 90% specific and had a positive predictive value of 88%, and negative predictive value of 90% for detection of spinal dural arterial venous fistulas [50]. Digital subtraction spinal angiography is considered the gold standard for identifying vascular abnormalities and is frequently used in evaluation of the bleeding source [40]. However, Braun et al. suggest performing spinal angiography when clinical suspicion of vascular malformation exists based on MRI findings [22]. In the present case, MRA demonstrated no vascular malformations; the patient had a poor and declining neurological examination requiring emergent surgical intervention, and no evidence of a malformation was noted intraoperatively. Given all these factors, clinical observation rather than a follow-up spinal digital subtraction angiograph was elected.

### 4.5. Treatment and Outcomes

Three treatment options exist in the management of sSDH: surgical evacuation, conservative medical management, and percutaneous drainage. If only mild deficits are present, conservative management is reasonable. However, in the face of clinical deterioration or severe motor/sensory deficits, surgical evacuation is advised [23]. Percutaneous drainage may be considered in cases where the hematoma is located dorsally and there is absence of coagulopathy [10, 15]. The current patient underwent urgent surgical decompression in light of the severe neurological deficits on initial presentation. Results from the literature review reveal that a greater proportion of patients experience complete or good neurological recovery when managed with conservative therapies alone (86%) versus those who underwent surgical interventions (47%). However, patients presenting with severe neurological deficits are more likely to receive surgical interventions, therefore introducing bias in favor of conservative management. In patients presenting with severe neurological deficits, urgent surgical decompression is indicated. Although when only modest neurological deficits are present, conservative therapies may be considered over surgical intervention.

The mortality rates in patients with spontaneous nontraumatic sSDH has decreased in recent years and is currently reported to be 1.3%. However, the associated morbidity, including serious neurologic deficits, is substantially higher and is reported to be 28% [1]. Pereira et al. examined factors that predict outcome in patients with spontaneous nontraumatic sSDH. Neurologic status at presentation was the strongest predictor of good outcomes; only 34% of patients with preexisting neurologic deficits had favorable outcomes compared to 83% of patients devoid of neurologic deficits at initial presentation. In the present case, the patient initially presented with severe neurologic dysfunction. Despite urgent surgical decompression, the patient has experienced limited recovery and persistent paraparesis. Other factors identified as predictive of favorable outcome include absence of coagulopathy, lumbar puncture, or other associated diseases. This may suggest outcomes in idiopathic cases will be more favorable as, by definition, they lack coagulopathies and iatrogenic factors. While the presence of subarachnoid hemorrhage has been implicated in theories regarding the etiology of spontaneous sSDH, the presence of subarachnoid hemorrhage was not found to be associated with outcome. Surgery was also not found to be associated with a more favorable outcome; however, Pereira et al. note a potential bias as patients in better clinical condition are less likely to receive surgical interventions.

## 5. Conclusion

Although rare, spontaneous sSDH should be considered in patients presenting with progressive motor, sensory, and autonomic deficits in addition to other intraspinal hematomas and inflammatory lesions. Although more common in patients with coagulopathies or following iatrogenic causes, sSDH can occur in the absence of an obvious underlying cause. The present case is illustrative of the substantial morbidity associated with the condition despite rapid diagnosis and surgical intervention. Due to the significant morbidity associated with the spontaneous sSDH, special consideration should be given to this diagnosis in patients with suggestive symptoms. Furthermore, surgical intervention is recommended in patients presenting with severe neurological deficits, although presence of these deficits is predictive of less favorable outcome. Conservative management should be strongly considered in patients with minor deficits as a large proportion of patients treated in this manner achieve favorable neurological recovery.

## Figures and Tables

**Table 1:** Summary of results from the literature review: Cases of spontaneous spinal subdural hematoma.

	Author and year	Age, years	Sex	Location	Presenting symptoms	Potential RFs	SAH	Treatment	Outcome
(1)	Ainslie, 1958 [5]	67	F	T8–T10	Back pain, paraperesis, bladder dysfunction	No	Yes	Laminectomy T8–T10	Complete recovery

(2)	Schaake and Schafer, 1970 [6]	74	M	NP	NP	NP	No	Surgery	Poor recovery

(3)	Anagnostopoulos and Gortvai, 1972 [7]	63	F	T8–T12	Back, arm, & abdominal pain, paraparesis, bladder dysfunction	No	No	Laminectomy T8–T12	Partial recovery

(4)	Reynolds and Turner, 1978 [8]	57	M	C4–C8	HA, hip pain, paraplegia, hypoesthesia, bowel dysfunction	No	No	Laminectomy C3–T1	Initial improvement then death (cardiopulmonary arrest)

(5)	Sakata and Kurihara, 1984 [9]	56	M	L2-S1	Back pain, paraparesis	Possible RA	No	Laminectomy L2-S1	Complete recovery

(6)	Swann et al., 1984 [10]	46	F	Thoraco-lumbar junction	HA, back pain w/radiation to BLE, paraparesis	No	No	Percutaneous drainage	Complete recovery

(7)	Martinez et al., 1987 [11]	64	M	T5-T6	Paraparesis, hypoesthesia of BLE	No	No	Laminectomy T5-T6	Partial recovery

(8)	Mavroudakis et al., 1990 [12]	38	M	T1-T2	Interscapular pain w/radiation to arm/nipple, paresthesia, HA, vomiting	No	Yes	Conservative	Complete recovery

(9)	Jacquet et al., 1991 [13]	51	M	T6–T8	Back pain, HA, fever, vomiting, slight opisthotonus	No	Yes	Laminectomy T5–T7	Complete recovery

(10)	Longatti et al., 1994 [14]	54	M	T5-L5	Back pain w/radiation to BLE & interscapular area, paraparesis, bladder dysfunction	HTN	No	Conservative	Complete recovery

(11)	Kang et al., 2000 [15, 16]	49	F	T5-L3	Back pain, paraparesis	No	No	Conservative	Complete recovery

(12)	Kuker et al., 2000 [16]	81	M	Mid T spine	Back pain, paraparesis bladder dysfunction	No	No	Surgery	Complete recovery

(13)	Kuker et al., 2000 [16]	56	F	Thoracolumbar	Paraparesis, bladder dysfunction	No	No	Surgery	Good recovery

(14)	Kirsch et al., 2000 [17]	42	M	Craniocervical junction	Paraplegia, bladder dysfunction	Suicide attempt with natural gas	No	Laminectomy T2–T5	No recovery

(15)	Kirsch et al., 2000 [17]	34	M	T1–T4	Midscapular pain, BLE paresthesia	No	No	Conservative	Complete recovery

(16)	Boukobza et al., 2001 [18]	74	M	T6-L4	Back pain, mild motor deficit in R LE	HTN	No	Conservative	Complete recovery

(17)	Maeda et al., 2001 [19]	29	F	T1–T4	HA, nausea, neck pain, paraplegia	No	No	Conservative	Partial recovery

(18)	Yamada et al., 2003 [20]	38	F	T1–T7	Interscapular pain, dysesthesia in BLE, bladder dysfunction, motor deficits in BLE	Postpartum, HTN	No	Conservative	Complete recovery

(19)	Thiex et al., 2005 [21]	78	M	T4–T11	Paraplegia, bladder dysfunction	No	No	R-sided hemilaminectomy; T5–T11	Death (due to another cause/not SDH)

(20)	Braun et al., 2007 [22]	76	F	Cervicothoracic	Back pain w/radiation to arms	No	No	Conservative	Complete recovery

(21)	Braun et al., 2007 [22]	72	F	Cervical t0 lumbar	Neck, pain, tetraparesis	No	No	Conservative	Complete recovery

(22)	Kyriakides et al., 2007 [23]	44	M	T2–T6	Back pain, paraplegia, bladder/bowel dysfunction	No	Yes	Laminectomy	Partial recovery

(23)	Kim et al., 2008 [24]	48	F	T1–T4	Paraplegia, bladder dysfunction	Fibromuscular dysplasia	No	Laminectomy T1–T4	No recovery

(24)	Montano et al., 2008 [25]	54	F	T6–T8	Back pain, bladder/bowel dysfunction, paraesthesia, hypoesthesia	Polycystic kidney disease	No	Surgery	Complete recovery

(25)	Ozdemir et al., 2008 [26]	50	M	T4–T8	Interscapular pain, paraparesis, hypoesthesia	No	No	Laminectomy T4–T6	Complete recovery

(26)	Al et al., 2009 [27]	57	M		HA, back pain, paraplegia, bladder/bowel dysfunction	No	No	Conservative	Complete recovery

(27)	Oh et al., 2009 [28]	59	F	C3–C6	Neck pain, L-sided hemiparesis	HTN, hyperlipidemia	No	Conservative	Complete recovery

(28)	Yang et al., 2009 [29]	35	F	L3-S1	HA, back pain, paraparesis	Concurrent intracranial SDH	No	Laminectomy	Complete recovery

(29)	Kakitsubata et al., 2010 [3]	66	M	T11-T12	HA, back pain, L LE pain	No	Yes	Conservative	Complete recovery

(30)	Nardone et al., 2010 [30]	37	M	C4-T4	HA, neck stiffness, cervical radicular pain, paraparesis, hypoesthesia	No	No	Conservative	Complete recovery

(31)	Liu et al., 2010 [31]	41	M	Mid T spine	Back pain, paraparesis, bladder dysfunction	Rhabdomyolysis, amphetamine abuse	No	Laminectomy T10-L1	Complete recovery

(32)	Nagashima et al., 2010 [32]	66	M	L1-S1	Leg pain, paraparesis, hypoesthesia, bowel dysfunction	Concurrent intracranial SDH, RA, HTN	No	Conservative	Complete recovery

(33)	Chung et al., 2011 [33]	45	F	T5–T11	Back pain, paraparesis, bladder dysfunction	HTN, DM	No	Conservative	Good recovery

(34)	Song et al., 2011 [34]	57	M	C1-T3	Neck & shoulder pain, paraparesis	Chronic renal failure, HTN	No	Conservative	Complete recovery

(35)	Yang et al., 2011 [35]	55	F	C2-T6	Paraplegia, hypoesthesia	HTN, DM	No	Conservative	Good recovery

(36)	Yang et al., 2011 [35]	38	M	C6-T5	HA, back pain, cold sweating, dizziness, vertigo, chest pain, hypoesthesia	No	No	Conservative	Good recovery

(37)	Cave and Sharobeem, 2013 [36]	65	M	T12	Back pain, paraplegia	No	No	Conservative	Partial recovery

(38)	Chung et al., 2014 [37]	66	F	C7-T4	HA, neck stiffness	No	No	Conservative	Complete recovery

(39)	Lin and Layman, 2014 [38]	70	M	L4-S1	Back pain, BLE weakness	HTN, hyperlipidemia, cancer, concurrent intracranial SDH	No	Conservative	Partial recovery

(40)	Oh and Eun, 2015 [39]	27	M	T5–T9	Back pain, paraparesis, hypoesthesia, bowel dysfunction, erectile dysfunction	No	No	Conservative	Good recovery

(41)	Visocchi et al., 2015 [40]	45	F	T1–T10	Back pain, paraplegia, anesthesia, bladder & bowel dysfunction	HIV+, HCV+, history of drug abuse	No	Laminectomy T1–T10	Partial recovery

(42)	Zhu et al., 2015 [41]	45	F	T9	Paraplegia, hypoesthesia	No	No	Laminectomy T8–T10	Partial recovery

(43)	Current case, 2017	43	M	T8–T11	Back pain, paraplegia	Drug abuse	No	Laminectomy T8–T10	Poor recovery

BLE: bilateral lower extremity; NP: data not provided; RA: rheumatoid arthritis; HTN: hypertension: L: left; DM: diabetes mellitus; SDH: subdural hematoma.

## References

[1] Pereira B. J., de Almeida A. N., Muio V. M., de Oliveira J. G., de Holanda C. V., Fonseca N. C. (2016). Predictors of Outcome in Nontraumatic Spontaneous Acute Spinal Subdural Hematoma: Case Report and Literature Review. *World Neurosurgery*.

[2] Domenicucci M., Ramieri A., Ciappetta P., Delfini R. (1999). Nontraumatic acute spinal subdural hematoma: report of five cases and review of the literature. *Journal of Neurosurgery*.

[3] Kakitsubata Y., Theodorou S. J., Theodorou D. J. (2010). Spontaneous spinal subarachnoid hemorrhage associated with subdural hematoma at different spinal levels. *Emergency Radiology*.

[4] Rader J. P. (1955). Chronic subdural hematoma of the spinal cord: report of a case. *The New England Journal of Medicine*.

[5] Ainslie J. P. (1958). Paraplegia due to spontaneous extradural or subdural haemorrhage. *British Journal of Surgery*.

[6] Schaake T., Schafer E. R. (1970). Spontaneous haemorrhage in the spinal canal.. *Journal of Neurology, Neurosurgery & Psychiatry*.

[7] Anagnostopoulos D. I., Gortvai P. (1972). Spontaneous Spinal Subdural Haematoma. *British Medical Journal*.

[8] Reynolds A. F., Turner P. T. (1978). Spinal subdural hematoma. *Rocky Mountain Medical Journal*.

[9] Sakata T., Kurihara A. (1984). Spontaneous spinal subdural hematoma. A case report. *The Spine Journal*.

[10] Swann K. W., Chung C. K., Kim H. J. (1984). Spontaneous spinal subdural hematoma with spontaneous resolution. *Spinal Cord*.

[11] Martinez R., Vaquero J., Gilsanz F. (1987). Spontaneous spinal subdural hematoma. Case report. *Journal of Neurosurgical Sciences*.

[12] Mavroudakis N., Levivier M., Rodesch G. (1990). Central cord syndrome due to a spontaneously regressive spinal subdural hematoma. *Neurology*.

[13] Jacquet G., Godard J., Orabi M., Sönmez S., Steimlé R. (1991). Spinal subdural hematoma. *Zentralblatt Fur Neurochirurgie*.

[14] Longatti P. L., Freschi P., Moro M., Trincia G., Carteri A. (1994). Spontaneous spinal subdural hematoma. *Journal of Neurosurgical Sciences*.

[15] Kang H.-S., Chung C.-K., Kim H. J. (2000). Spontaneous spinal subdural hematoma with spontaneous resolution. *Spinal Cord*.

[16] Kuker W., Thiex R., Friese S. (2000). Spinal subdural and epidural haematomas: diagnostic and therapeutic aspects in acute and subacute cases. *Acta Neurochir (Wien)*.

[17] Kirsch E. C., Khangure M. S., Holthouse D., McAuliffe W. (2000). Acute spontaneous spinal subdural haematoma: MRI features. *Neuroradiology*.

[18] Boukobza M., Haddar D., Boissonet M., Merland J. J. (2001). Spinal subdural haematoma: a study of three cases. *Clinical Radiology*.

[19] Maeda M., Mochida J., Toh E., Nishimura K., Nomura T. (2001). Nonsurgical treatment of an upper thoracic spinal subdural hemorrhage. *Spinal Cord*.

[20] Yamada K., Nakahara T., Yamamato K., Muranaka T., Ushio Y. (2003). Nontraumatic spinal subdural haematoma occurring in a postpartum period. *Acta Neurochir (Wien)*.

[21] Thiex R., Thron A., Gilsbach J. M., Rohde V. (2005). Functional outcome after surgical treatment of spontaneous and nonspontaneous spinal subdural hematomas. *Journal of Neurosurgery: Spine*.

[22] Braun P., Kazmi K., Nogués-Meléndez P., Mas-Estellés F., Aparici-Robles F. (2007). MRI findings in spinal subdural and epidural hematomas. *European Journal of Radiology*.

[23] Kyriakides A. E., Lalam R. K., El Masry W. S. (2007). Acute spontaneous spinal subdural hematoma presenting as paraplegia: A rare case. *The Spine Journal*.

[24] Kim S. D., Park J. O., Kim S. H., Lee Y. H., Lim D. J., Park J. Y. (2008). Spontaneous thoracic spinal subdural hematoma associated with fibromuscular dysplasia. *Journal of Neurosurgery: Spine*.

[25] Montano N., Nucci C. G., Doglietto F. (2008). Teaching NeuroImage: Spontaneous idiopathic spinal subdural hematoma. *Neurology*.

[26] Ozdemir O., Calisaneller T., Yildirim E., Caner H., Altinors N. (2008). Acute spontaneous spinal subdural hematoma in a patient with bilateral incarcerated inguinal hernia. *Joint Bone Spine*.

[27] Al B., Yildirim C., Zengin S., Genc S., Erkutlu I., Mete A. (2009). Acute spontaneous spinal subdural haematoma presenting as paraplegia and complete recovery with non-operative treatment. *BMJ Case Reports*.

[28] Oh S. H., Han I., Koo Y., Kim O. (2009). Acute Spinal Subdural Hematoma Presenting with Spontaneously Resolving Hemiplegia. *Journal of Korean Neurosurgical Society*.

[29] Yang M. S., Tung Y. W., Yang T. H. (2009). Spontaneous spinal and intracranial subdural hematoma. *Journal of the Formosan Medical Association*.

[30] Nardone R., Kunz A., Kraus J. (2010). Spontaneous subdural spinal haematoma presenting as occipital headache: a case report. *Acta Neurologica Belgica*.

[31] Liu C., Cheng C., Cho D. (2010). Rhabdomyolysis Accompanied by Spontaneous Spinal Subdural and Subarachnoid Hematoma Related to Amphetamine Abuse. *The Spine Journal*.

[32] Nagashima H., Tanida A., Hayashi I. (2010). Spinal subdural haematoma concurrent with cranial subdural haematoma: Report of two cases and review of literature. *British Journal of Neurosurgery*.

[33] Chung T. T., Cheng-Ta H., Ming-Ying L., Da-Tong J. (2011). Spontaneous spinal subdural hematoma: A rare case report and review of the literature. *Journal of Medical Sciences*.

[34] Song T. J., Lee J. B., Choi Y. C., Lee K. Y., Kim W. J. (2011). Treatment of spontaneous cervical spinal subdural hematoma with methylprednisolone pulse therapy. *Yonsei Medical Journal*.

[35] Yang N.-R., Kim S. J., Cho Y. J., Cho D. S. (2011). Spontaneous resolution of nontraumatic acute spinal subdural hematoma. *Journal of Korean Neurosurgical Society*.

[36] Cave J. J., Sharobeem K. M. (2013). A rare case of spontaneous spinal subdural haematoma that developed after using an electric drill. *Cerebrovascular Disease*.

[37] Chung J., Park I. S., Hwang S., Han J. (2014). Acute Spontaneous Spinal Subdural Hematoma with Vague Symptoms. *Journal of Korean Neurosurgical Society*.

[38] Lin J. C., Layman K. (2014). Spontaneous spinal subdural hematoma of intracranial origin presenting as back pain. *Journal of Emergency Medicine*.

[39] Oh Y. M., Eun J. P. (2015). Idiopathic spontaneous spinal subdural hematoma causing transient paraparesis: Case report with a review of the literature. *Neurosurgery Quarterly*.

[40] Visocchi M., La Rocca G., Signorelli F., Roselli R., Jun Z., Spallone A. (2015). 10 Levels thoracic no-intrumented laminectomy for huge spontaneous spinal subdural hematoma removal. report of the first case and literature review. *International Journal of Surgery Case Reports*.

[41] Zhu Y. J., Peng D. Q., Shen F., Wang L. L. (2015). Spontaneous thoracic ventral spinal subdural hematoma mimicking a tumoral lesion: a case report. *Journal of Medical Case Reports*.

[42] Sokoloff J., Coel M. N., Ignelzi R. J. (1976). Spinal subdural hematoma. *Radiology*.

[43] Hirano K., Tada M., Sasahira N. (2014). Incidence of malignancies in patients with IgG4-related disease. *Internal Medicine*.

[44] Zochodne D., Hinton G., Del Maestro R. (1986). Intradural spinal hematoma in an infant with cystic fibrosis. *Pediatric Neurology*.

[45] Pujol S., Torrielli R. (1996). Neurological accidents after epidural anesthesia in obstetrics. *Cahiers D'Anesthesiologie*.

[46] Lao T. T., Halpern S. H., MacDonald D., Huh C. (1993). Spinal subdural haematoma in a parturient after attempted epidural anaesthesia. *Canadian Journal of Anesthesia*.

[47] Edelson R. N., Chernik N. L., Posner J. B. (1974). Spinal Subdural Hematomas Complicating Lumbar Puncture. *JAMA Neurology*.

[48] Russell N., Benoit B. (1983). Spinal subdural hematoma a review. *World Neurosurgery*.

[49] Buxton N., McConachie N. S. (2016). Amphetamine abuse and intracranial haemorrhage. *Journal of the Royal Society of Medicine*.

[50] Saindane A. M., Boddu S. R., Tong F. C., Dehkharghani S., Dion J. E. (2015). Contrast-enhanced time-resolved mra for pre-angiographic evaluation of suspected spinal dural arterial venous fistulas. *Journal of NeuroInterventional Surgery*.

